# A Case Report of Severe Posterior Reversible Encephalopathy Syndrome Due to Accelerated Hypertension in a Young Patient

**DOI:** 10.7759/cureus.26918

**Published:** 2022-07-16

**Authors:** Suhrim Choe, Nagapratap Ganta, Dina Alnabwani, Sharon Hechter, ghadier Alsaoudi, Vraj Patel, Ankita Prasad, Pramil Cheriyath

**Affiliations:** 1 Internal Medicine, Hackensack Meridian Health (HMH) Ocean Medical Center, Brick, USA; 2 internal Medicine, Jersey Shore University Medical Center, Neptune, USA; 3 Internal Medicine, Hackensack Meridian Health (HMH) Ocean Medical Center, brick, USA; 4 Internal Medicine, Hackensack Meridian Health (HMH) Ocean University Medical Center, Brick, USA

**Keywords:** pres in transplant, uncontrolled hypertension, cerebellar herniation, posterior cerebral edema, vasogenic edema, posterior reversible encephalopathy syndrome (pres)

## Abstract

Posterior reversible encephalopathy syndrome (PRES) refers to white matter vasogenic edema primarily affecting the brain's posterior occipital and parietal lobes, causing acute neurological symptoms like headaches, visual symptoms, seizures, and altered mental status. We present the case of a 32-year-old male with uncontrolled hypertension, altered mental status, and left-sided weakness. He had a rapid neurological decline, and a computed tomography (CT) head showed blurring of gray-white matter interfaces in the right posterior parietal lobe, suggesting infarction or PRES. Magnetic resonance imaging (MRI) of the brain suggested worsening with acute-early subacute infarction involving the right temporal, parietal, and occipital lobes and diffuse cerebral edema causing compression of the right ventricle with diffuse sulcal effacement and central downward herniation. There were flair hyperintensities in the bifrontal, pons, and cerebellum. Given the history of uncontrolled hypertension, the right hemispheric infarction and edema were thought to be due to secondary complications of severe PRES. He underwent urgent bilateral craniectomies with dural augmentation and external ventricular drain placement to control the intracranial pressure the next day. His mental status, as well as neurologic function, showed gradual improvement in the next few months. A high index of suspicion and rapid treatment can pave the way for a quick recovery and help reduce morbidity and death.

## Introduction

Posterior reversible encephalopathy syndrome (PRES) is an acute neurological condition with many radiological features. Common neurological complaints include headaches, loss of consciousness, seizures, vision symptoms, and localized neurological impairments. Labile blood pressure, renal failure, eclampsia, exposure to immunosuppressive or cytotoxic drugs, and autoimmune illnesses [1] are the most common causes. History, clinical examination, and radiologic findings of symmetric bilateral hyper-intensities on T2-weighted magnetic resonance imaging (MRI) reflecting vasogenic edema are used to diagnose PRES [2]. The pathophysiology of PRES is uncertain, and numerous mechanisms have been proposed. Many of these mechanisms can be present together, like hyper-perfusion due to loss of autoregulatory vascular tone, hypoperfusion due to systemic vasoconstriction, and malfunction or endothelial injury due to a blood-brain barrier lesion. The presence of white matter vasogenic edema involving the posterior cerebral lobes (parietal and occipital lobes) is seen in MRI of the brain [3]. PRES is reported in up to 2.7-25% of bone marrow transplantation patients, 0.4% of solid organ transplantation patients, 0.84% of patients with end-stage renal disease, and 0.69% of patients with systemic lupus erythematosus [1]. PRES was an underdiagnosed neurological disorder, but because of the increased availability and higher quality of imaging, awareness of the disease has markedly improved.

## Case presentation

A 32-year-old male with a past medical history of transient ischemic attacks, hypertension, non-compliance with medication, and alcohol use disorder presented with altered sensorium and transient left-sided weakness to the emergency room. He had similar symptoms four months ago and left the hospital against medical advice. This time, he had a headache and excessive sleepiness for three days. His family called emergency medical services when he was found staring blankly, not responding to verbal stimuli, and not moving his left upper and lower limbs. On the way to the hospital, his left-sided weakness and sensorium improved. His family history had significant hypertension in his father. He is a heavy smoker, uses marijuana occasionally, and drinks 10-12 beers weekly, but his family denied recent alcohol use. At the presentation, he was somnolent and not oriented. He had an elevated blood pressure of 224/113 mm Hg; his heart rate was 64 beats per minute, and his SpO2 was 96% on room air. On a neurological examination, he had no extraocular movements on the left side and could not blink. Muscle strength was 3/5 in the left upper and lower extremities.

**Figure 1:**
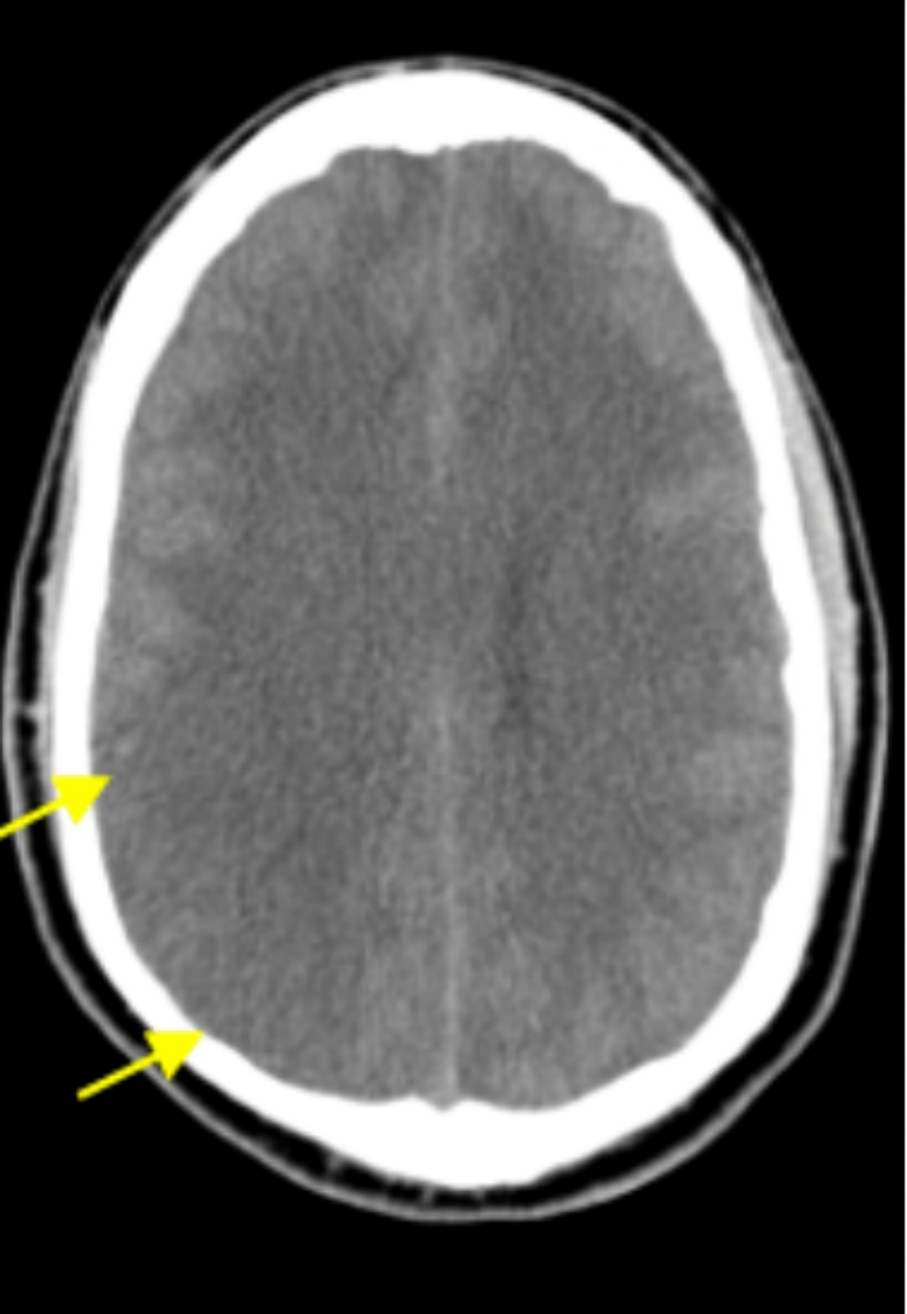
CT brain showing loss of gray white differentiation in parietal lobe suggesting infarct which may be due to PRES

Significant lab findings were raised: blood urea nitrogen (BUN) of 33 mg/dL, serum creatinine of 2.48 mg/dL, and a decreased glomerular filtration rate (markedly diminished compared to the previous GFR measured in March of 2021 was 59 mL/min), high anion gap of 15 mmol/L, elevated troponin of 0.14 ng/mL and elevated total bilirubin of 2.3 mg/dL. Other blood work, including blood glucose and serum electrolytes, was normal. Initial head CT showed blurring of the gray-white matter interface in the right posterior parietal lobe (Figure 1).

**Figure 2:**
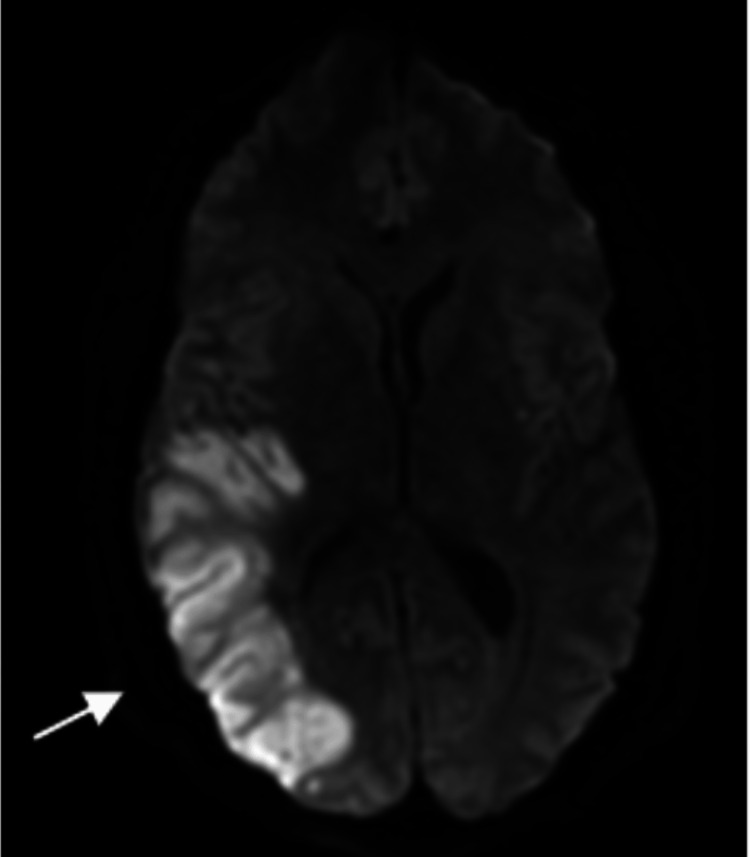
MRI brain diffusion-weighted imaging series showing restricted diffusion in temporal, parietal, occipital regions

**Figure 3:**
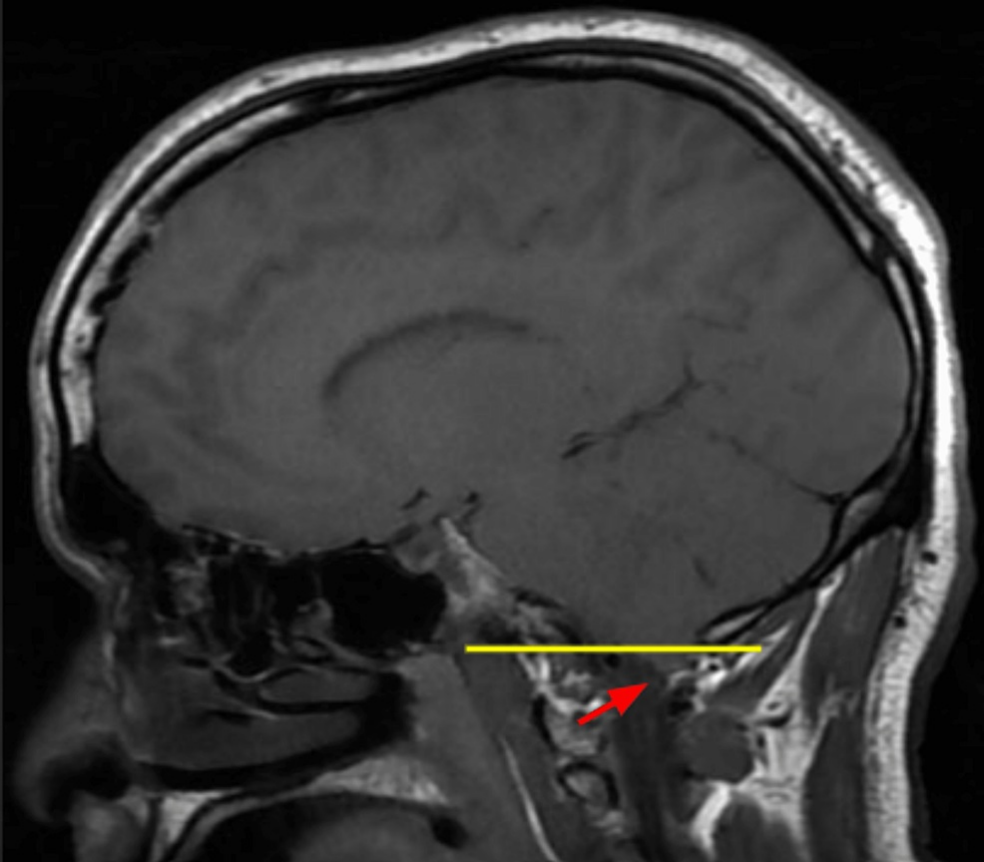
MRI brain T1 weighted series showing cerebellar tonsils herniated below foramen magnum

**Figure 4:**
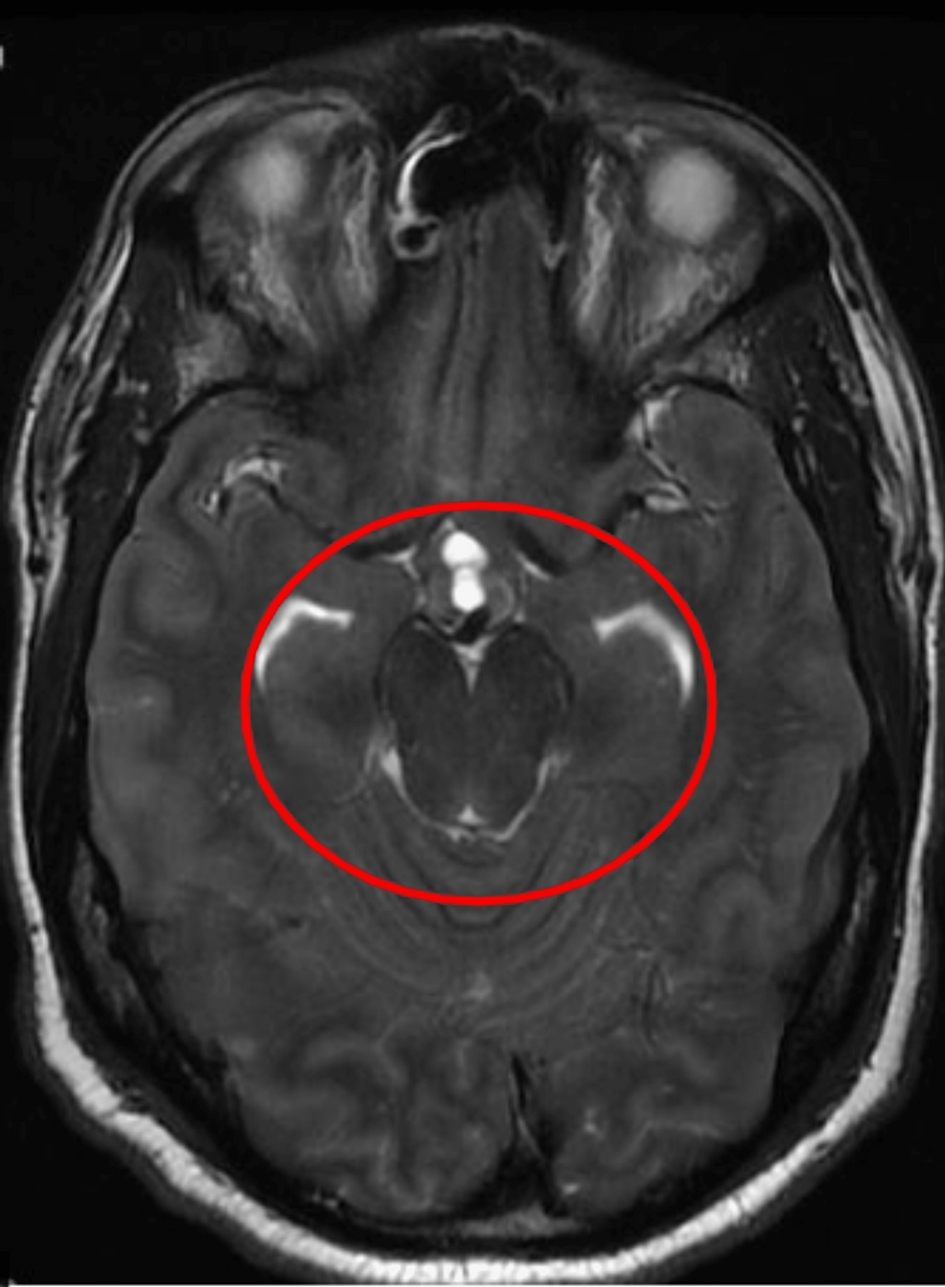
MRI brain T2 weighted series showing crowding of the cisterns suggesting herniation

**Figure 5:**
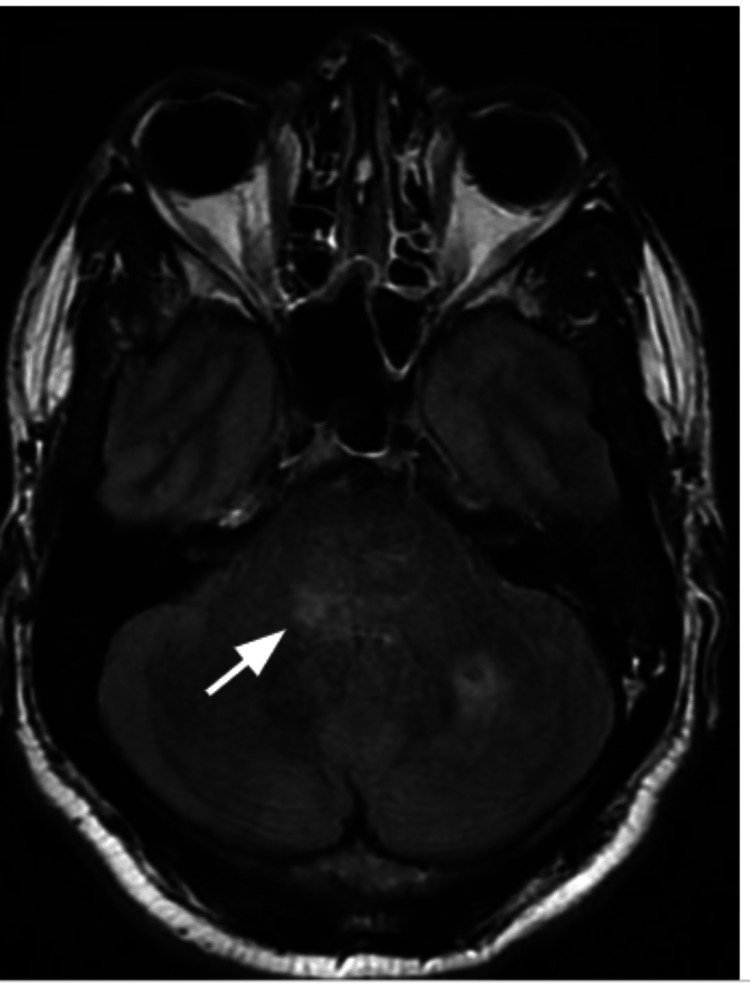
MRI brain FLAIR T2 weighted series showing edema in the pons

**Figure 6:**
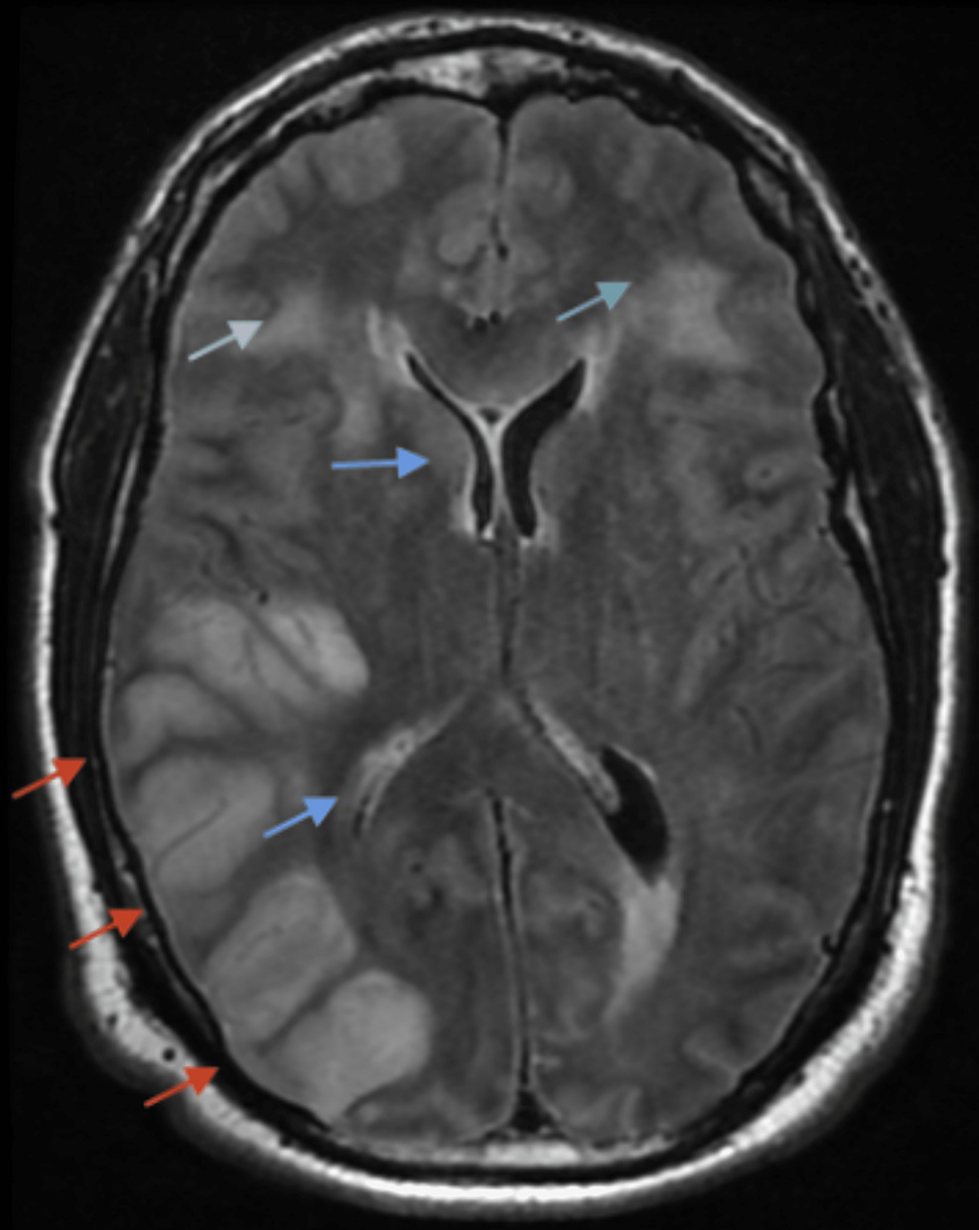
MRI brain FLAIR T2 weighted series showing diffuse sulcal edema in the temporal, parietal, occipital regions (red arrow), effacement of the right lateral ventricle (blue arrow), and bifrontal edema (green arrow)

Of note, an MRI of the brain without contrast from four months prior to this admission showed moderate white matter change involving the corpus callosum, some of which were oriented perpendicular to the ventricle, and a new small hyperintense lesion just posterior to the pineal gland. The MR angiogram of the neck with and without contrast from the same time showed tortuosity of the high cervical internal carotid arteries, which can be seen in vasculopathy, such as fibromuscular dysplasia, connective tissue disorders, and long-standing uncontrolled hypertension. A chest X-ray at admission showed minimal pleural effusion in the left lung with adjacent atelectasis, mild pulmonary congestion, and an enlarged heart shadow. His blood pressure was managed with labetalol and hydralazine on day one, but on the second day, he was found unresponsive with a blood pressure of 220/118 mm/Hg. A Nicardipine drip was started, and he was transferred to the intensive care unit (ICU). Repeat CT head (without contrast) showed increased blurring of gray-white matter interfaces in the right posterior parietal lobe, suggesting infarct or PRES. His blood pressure continued to be labile, with systolic blood pressure fluctuating between 140 and 200 mmHg despite maximum doses of IV Nicardipine continuous drip, IV labetalol, clonidine patch, and IV hydralazine. His mental status showed improvement during this time, and he started following commands and answering simple questions appropriately. However, he soon had an acute change in mental and neurologic status, demonstrating stupor, slow response to noxious stimuli, and 4-5 mm dilated pupils in both eyes with no corneal reflex on the right. The National Institute of Health (NIH) stroke scale was at 25 during this time, much worse than the NIH stroke scale of 15 a few hours prior. He had a brief clonic movement on the right side of his body. He was intubated, and a repeat CT and MRI of the head (Figure 2) showed continued evolution of right parietal edema with complete effacement of the right posterior horn, which may represent an acute or subacute infarct with associated cytotoxic edema and blurring of the gray-white matter interface in the right posterior parietal lobe, suggesting infarct or PRES. He received one dose of mannitol and 3% hypertonic saline; IV continuous propofol was started. Subsequent MRI of the brain from the same day also corroborated the worsening disease demonstrated by the acute-early subacute infarction involving the right temporal, parietal, and occipital lobes and associated mass effects, including compression of the right ventricle and diffuse sulcal effacement and evidence of central downward herniation suggestive of diffuse cerebral edema. In addition, MRI also demonstrated flair hyperintensities in the bifrontal, pons, and cerebellum (Figures 3-6).

Given the possibility of PRES, the right hemisphere infarction with malignant edema was suspected to be due to secondary complications of severe PRES. He underwent urgent craniectomy with dural augmentation and external ventricular drain placement to control the intracranial pressure suggestive of diffuse cerebral edema. In addition, MRI also demonstrated flair hyperintensities in the bifrontal, pons, and cerebellum.

**Figure 7:**
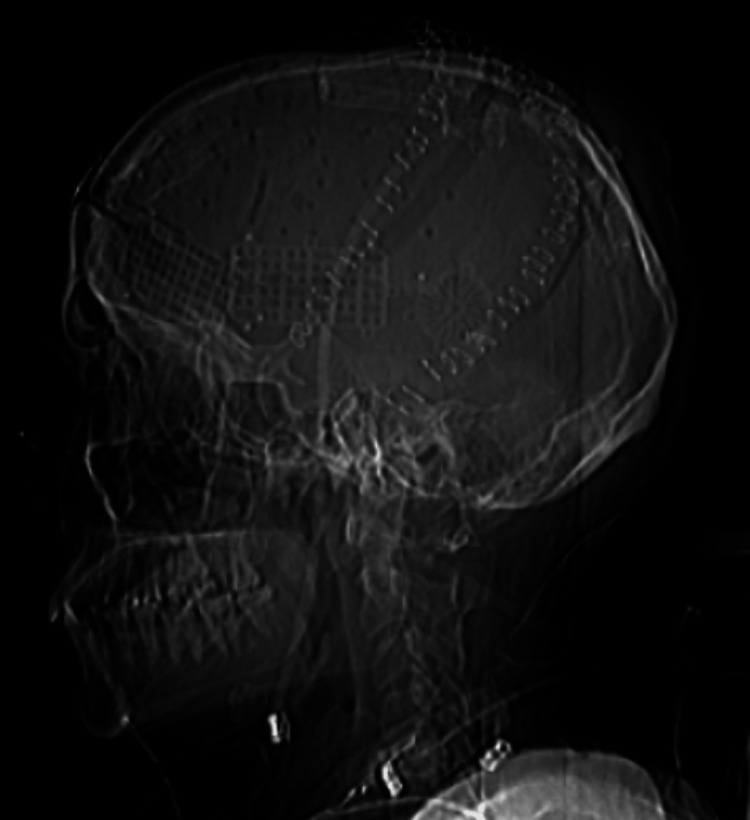
Status post bilateral cranioplasty

Approximately one month after the craniectomy, GFR was >60 mL/min, with a BUN of 39 mg/dL, magnesium of 2.5 mg/dL, and total protein of 7.8 mg/dL. He was discharged to a rehabilitation facility in stable condition for further improvement in functional status. His laboratory values, including renal function, gradually improved over a few months after the surgery. His mental and neurological status also improved. Follow-up CT of the head after four months showed a mild decrease in lateral and third ventricle size, and there was no loss of gray-to-white differentiation (Figure 7).

## Discussion

Hinchey et al. first described PRES in a series of 15 individuals in 1996 as the Reversible Posterior Leukoencephalopathy Syndrome [4]. PRES, a reversible neurological condition, involves white matter edema involving the occipital and parietal lobes. It has been documented in practically all age groups, from children to the elderly, but it is more common in young and middle-aged individuals, with female predominance [5]. While the incidence of PRES in the general population is unknown, it has been documented in a small group of patients. It has been recorded in 2.7-25% of patients after bone marrow transplantation, 0.4% after solid organ transplantation, 0.84% of patients with end-stage renal illness, and 0.69% with systemic lupus erythematosus among hospitalized adults [1]. In the hospitalized pediatric population, the incidence of PRES is 0.04%, and 0.4% in pediatric intensive care units.

The actual pathophysiological process behind PRES is unknown. To date, three explanations have been proposed: (1) cerebral vasoconstriction resulting in brain infarcts, (2) cerebral auto-regulation failure with vasogenic edema, and (3) endothelial injury with blood-brain barrier disturbance resulting in fluid and protein transudation in the brain [6]. The "vasogenic theory," which proposes that rapidly increasing hypertension combined with a lack of cerebral autoregulation leads to a breach of the blood-brain barrier and secondary vasogenic edema, is the most popular. The auto-regulating response is insufficient when blood pressure rises rapidly and dramatically, resulting in hyperperfusion and extravasation of plasma and macromolecules. The posterior circulation of the brain is comparably less innervated with sympathetic nerves, which is most likely the cause of PRES’s preferential involvement of the posterior portion of the brain [7].

‌PRES is characterized by a wide range of neurological symptoms that generally occur in the setting of elevated arterial blood pressure. Cases of PRES were first reported in patients with hypertension and later observed in normotensive and septic patients. Acute blood pressure elevation, renal insufficiency, preeclampsia/eclampsia, autoimmune disorders, infection, transplantation, and chemotherapeutic medications are all common risk factors for PRES [1]. PRES can be acute or subacute, with neurologic symptoms that may appear from a few hours to weeks [5]. Quantitative and qualitative illnesses of consciousness, including cognitive impairment, stupor, somnolence, or coma, may be manifested as signs of encephalopathy. Epileptic seizures, reported in roughly two-thirds of all PRES patients, can be focal and generalized [5,8,9]. Visual disturbances such as visual field abnormalities, including hemianopia and cortical blindness, worsening of visual acuity, or visual hallucinations, can be seen in approximately two-thirds of all PRES patients owing to the frequent involvement of the occipital lobes [4,5]. A few myelopathic symptoms and cases demonstrating spinal cord involvement have been reported. Other rare clinical manifestations include abulia, agitation, delusions, opisthotonus, optic ataxia, ocular apraxia, and simultagnosia [1].

The following criteria were proposed by Fugate and Schmutzhard for the diagnosis of PRES: sudden onset of neurological symptoms, abnormalities on neuroimaging suggesting (focal) vasogenic edema, and the reversibility of clinical and radiological findings [5]. Comorbidities or triggering factors in the clinical context and the presence of acute onset neurological symptoms, concurrent labile blood pressure, and vasogenic edema reflected on neuroimaging findings suggest PRES. The cornerstone of confirming a diagnosis of PRES is brain imaging. Although non-contrast computed tomography (CT) can detect vasogenic edema in some cases, brain MRI, particularly the T2-weighted and fluid-attenuated inversion recovery (FLAIR) sequences, is far more sensitive [10]. The traditional imaging patterns usually demonstrate bilateral, subcortical, and symmetrical vasogenic edema involving the parieto-occipital region. Other than the parietal occipital pattern, the holohemispheric watershed and superior frontal sulcus pattern are different patterns described in the literature [1]. Differential diagnoses of PRES include cerebrovascular accidents, meningoencephalitis, demyelinating disorders of the brain, and cerebral venous thrombosis. Early imaging is the key to making the diagnosis [11].

Because there is no specific management strategy currently available for PRES, it is treated symptomatically. The treatment of the underlying condition that leads to the development of PRES is critical. Eliminating the triggering factor or treating the underlying pathology should be initiated as soon as possible in the course of the disease [5]. Supportive care should be provided, including hydration, correction of electrolyte imbalance, airway monitoring, and ventilation support, especially for patients with altered mental status. Prompt dialysis is recommended for patients with renal failure. For patients in hypertensive crisis, no more than 20-25% of the blood pressure should be decreased in the first few hours to reduce the risk of cerebral, coronary, and renal ischemia [1]. Since the neurological symptoms of PRES are largely reversible in most patients, the prognosis is mainly dictated by underlying conditions. Although reversible, secondary complications, including massive ischemic infarction, intracranial hemorrhage, and status epilepticus, may lead to permanent neurologic deficits and death. Although inciting factors commonly recur, the recurrence of PRES has been recorded to be infrequent (less than 10%) [11].

## Conclusions

PRES is a reversible illness that is defined by acute neurologic symptoms and radiographic evidence of vasogenic edema, typically involving parieto-occipital areas. Acute to subacute neurologic symptoms including confusion, visual symptoms, headache, and posterior transitory alterations on neuroimaging are used to identify PRES. Treatment is primarily determined by the underlying disease, and success is dependent on early detection and treatment of the underlying condition. Early detection and treatment of this uncommon condition are critical for decreasing the risk of persistent neurologic impairments and death.
